# Comparison of false-negative/positive results of intraoperative evoked potential monitoring between no and partial neuromuscular blockade in patients receiving propofol/remifentanil-based anesthesia during cerebral aneurysm clipping surgery: A retrospective analysis of 685 patients

**DOI:** 10.1097/MD.0000000000004725

**Published:** 2016-08-26

**Authors:** Sung-Hoon Kim, Seok-Joon Jin, Myong-Hwan Karm, Young-Jin Moon, Hye-Won Jeong, Jae-Won Kim, Seung-Il Ha, Joung-Uk Kim

**Affiliations:** Department of Anesthesiology and Pain Medicine, Asan Medical Center, University of Ulsan College of Medicine, Seoul, Korea.

**Keywords:** aneurysm, electrophysiology, neuromuscular blockade, neurosurgery, remifentanil

## Abstract

Although the elicited responses of motor evoked potential (MEP) monitoring are very sensitive to suppression by anesthetic agents and muscle relaxants, the use of neuromuscular blockade (NMB) during MEP monitoring is still controversial because of serious safety concerns and diagnostic accuracy. Here, we evaluated the incidence of unacceptable movement and compared false-negative MEP results between no and partial NMB during cerebral aneurysm clipping surgery. We reviewed patient medical records for demographic data, anesthesia regimen, neurophysiology event logs, MEP results, and clinical outcomes. Patients were divided into 2 groups according to the intraoperative use of NMB: no NMB group (n = 276) and partial NMB group (n = 409). We compared the diagnostic accuracy of MEP results to predict postoperative outcomes between both groups. Additionally, we evaluated unwanted patient movement during MEP monitoring in both groups. Of the 685 patients, 622 (90.8%) manifested no intraoperative changes in MEP and no postoperative motor deficits. Twenty patients showed postoperative neurologic deficits despite preserved intraoperative MEP. False-positive MEP results were 3.6% in the no NMB group and 3.9% in the partial NMB group (*P* = 1.00). False-negative MEP results were 1.1% in the no NMB group and 4.2% in the partial NMB group (*P* = 0.02). No spontaneous movement or spontaneous respiration was observed in either group. Propofol/remifentanil-based anesthesia without NMB decreases the stimulation intensity of MEPs, which may reduce the false-negative ratio of MEP monitoring during cerebral aneurysm surgery. Our anesthetic protocol enabled reliable intraoperative MEP recording and patient immobilization during cerebral aneurysm clipping surgery.

## 1 Introduction

Intracranial aneurysm surgery can cause cerebral infarction in the major cerebral arteries supplying the cortex and subcortical structures or in the perforating arteries feeding deep subcortical structures. Postoperative motor deficits caused by blood flow insufficiency are especially serious problems in cerebral aneurysm surgery. Early ischemia detection through electrophysiologic monitoring might lead to interventions, including unclipping or repositioning of surgical clip, preventing infarction.^[1]^ Methods for detecting intraoperative cerebral ischemia during intracranial clipping surgery should be sensitive and specific, and intraoperative monitoring of transcranial motor evoked potential (MEP) may improve neurologic outcome.^[2]^ However, a limitation of this monitoring technique is the percentage of false-negative MEP results reported in 5.5%^[3]^ or up to 40% of patients.^[4,5]^ As elicited responses of MEP monitoring are very sensitive to suppression by anesthetic agents and muscle relaxants, appropriate choice of anesthetic regimen is of importance to enhance the diagnostic accuracy of MEP monitoring.

In terms of MEP amplitude and variability, no neuromuscular blockade (NMB) is more desirable than various levels of partial NMB.^[6]^ Nevertheless, patient movement during MEP monitoring may interfere with surgery and raise serious safety concerns.^[7]^ Those who advocate partial NMB insist that the complete omission of NMB could result in problems such as unexpected patient movement.^[5,8,9]^ In aneurysm surgery, reported incidences of unacceptable movement during MEP with partial or no NMB are 3.2% to 7.5%.^[6,10]^ Studies have also shown that in 6% to 10% of patients undergoing MEP monitoring without NMB, recording was impossible because of electrostimulation-induced patient movement.^[11,12]^ Anesthesia for cerebral aneurysm clipping surgery combined with MEP monitoring should not only provide the best conditions for MEP monitoring, but also guarantee patient immobilization. However, a detailed anesthetic protocol to simultaneously provide the best MEP result and patient immobilization is still unclear.

To this purpose, we evaluated diagnostic accuracy of MEP monitoring to predict postoperative outcomes between no and partial NMB during cerebral aneurysm clipping surgery. In addition, we also assessed patient safety-related parameters, such as spontaneous movement or spontaneous respiration during MEP monitoring.

## 2 Patients and methods

The study was approved by the Institutional Review Board of Asan Medical Center, and the requirement for individual consent was waived. The electronic neurophysiology event log and medical records of all patients who underwent intracranial clipping surgery between January 2013 and December 2014 at our institution were retrospectively reviewed. Medical records included patient characteristics, aneurysm location, anesthetic regimen, and clinical outcomes. A standardized protocol was implemented for neurophysiologic monitoring during cerebral aneurysm clipping for departmental quality management. The neurophysiology event logs documented any change in stimulation intensity, recorded the reason for the change, and explained any inability to acquire MEP once baseline signals were obtained. All procedures were performed by 2 neurosurgeons and a limited number of experienced anesthesiologists. MEP monitoring was performed by trained and certified neurophysiologists. The clinical outcome of motor function was postoperatively reviewed for all patients by the attending anesthesiologist and neurosurgeon. Motor function was first reviewed in the operating room after tracheal extubation, in the presence of the neurosurgeon. In addition, patients were routinely evaluated after transfer to the postoperative intensive care unit and before relocation to a ward. In case of any motor deficit, patients were also evaluated every following day until recovery of the deficit or until discharge from the hospital. All patients showing intraoperative changes on MEP monitoring or having postoperative neurologic deficits (PNDs) were formally examined by a neurologist. Recovery from PNDs before hospital discharge was ranked as transient. Any motor deficit that persisted at the time of discharge was considered permanent for our purposes.

### 2.1 Anesthesia regimen

In all patients, standard American Society of Anesthesiologists monitors were applied. Before anesthesia induction, fentanyl (50–100 μg) was administered. After lidocaine local infiltration, an intra-arterial catheter was placed for invasive arterial pressure monitoring. Following the injection of lidocaine (20–40 mg), anesthesia was induced with propofol (1.5–2.5 mg/kg) via intravenous administration. A commercially available 2-channel target-controlled infusion (TCI) pump (Orchestra, Fresenius Vial, Brezins, France) was used for effect-site TCI of propofol and remifentanil. Endotracheal intubation was facilitated by a single bolus of rocuronium (0.8–1.0 mg/kg) or, when indicated, succinylcholine (0.8–1.2 mg/kg). At the discretion of the anesthesiologist, rocuronium was administered only once to provide neuromuscular blockade for endotracheal intubation, or rocuronium was continuously infused under train-of-four (TOF) monitor. Before rocuronium administration, the baseline twitch response was established with a neuromuscular transmission module (M-NMT Module, Datex-Ohmeda Inc., Helsinki, Finland). This module automatically searched for the stimulus current to achieve the maximal response of the adductor pollicis. The maximal electromyographic amplitude of T1 before rocuronium infusion was considered as the control response. All patients in partial NMB group were to maintain a 0.5 twitch height of the first evoked response of TOF stimulation (T1) compared with the control twitch. Before dural opening, anesthesia was maintained using continuous infusion of propofol (2.5 μg/mL) and remifentanil (7–9 ng/mL) for total intravenous anesthesia (TIVA). After dural opening, TCI rates were increased to 3.0 μg/mL for propofol and 10 to 15 ng/mL for remifentanil. Bispectral index (Aspect Medical Systems Inc., Framingham, MA) was used to evaluate adequate depth of anesthesia (target index range, 30–50). The patient was ventilated with an air/oxygen mixture (fraction of inspired oxygen, 0.5), and ventilation was adjusted to achieve an arterial carbon dioxide pressure of 28 to 32 mm Hg. The target arterial systolic blood pressure during cerebral aneurysm clamping was set at 10% more than the preoperative values. In case of arterial hypotension, continuous infusion of phenylephrine was administered. Temperature adjustment was performed to achieve normothermia at the time of permanent aneurysm clip placement. Specific notes of patient movement were routinely made in the neurophysiology event log, followed by a temporary suspension of MEP monitoring or adjustment of MEP stimulation intensity. It is standard practice at our institution to document and explain any interruption of MEP monitoring or any change in MEP stimulation intensity. The neurophysiology event log should be a high-fidelity record of patient movement during aneurysm surgery.^[10]^ Patient movements included nociception-induced movement (defined as reaching for the endotracheal tube, coughing, chewing, or bucking) and excessive field movement (defined as gross visible head movement as identified by the surgical and anesthesia teams). Spontaneous respiration was closely monitored and was reported by the attending anesthesiologist.

### 2.2 MEP monitoring and evaluation of rates of success and changes in MEP monitoring

After anesthesia induction, the stimulating and recording electrodes were placed and baseline recordings for neurophysiologic monitoring were obtained. For recording MEP waves, a Digitimer Multi-Pulse Cortical Stimulator model-D185 (Digitimer Ltd, Hertfordshire, England) and Neuropack MEB-2200 (NIHON KOHDEN, Tokyo, Japan) were used. Stick-on type electrodes were placed subcutaneously at C3/C4 for left and right hemispheric stimulation (according to the International 10–20 electroencephalogram system). MEP was elicited using a train of 6 constant-current anodal 0.5-ms-wide stimuli delivered at 3-ms interstimulus intervals. Stimulus intensity was adjusted until evoked potential responses were detectable in the lower extremities. Bilateral MEPs were recorded using pairs of patch electrodes at the tibialis anterior, adductor hallucis, and the abductor pollicis brevis muscles. MEP was recorded within a 150-ms interval, filtered (band pass, 30–2000 Hz), and amplified (10,000 times). MEP was routinely recorded at dural opening, before vessel occlusion, during permanent clip placement, and at dural closing; MEP was recorded more frequently during critical surgical manipulations. Once the baseline signal was obtained, any change in stimulation intensity was documented in the neurophysiology event log. A change in MEP monitoring was defined as significant when the following EP alterations occurred: (1) MEP amplitude decrease of more than 50%, or (2) a loss of MEP in 3 consecutive trials. Video monitoring was used to assist the electrophysiology team in performing timed stimulations in coordination with the surgery. Additionally, somatosensory EP responses from both the median and tibial nerves were monitored, but these data were not the subject of the present study.

### 2.3 Statistical analysis

The rate of false-negative/false-positive results with MEP measurements was calculated and compared with the immediate postoperative outcome of motor function. A false-negative result was defined as the occurrence of immediate postoperative motor deficit without significant intraoperative changes in the EP monitor. A false-positive result was defined as the absence of postoperative motor deficit with significant intraoperative changes in the EP monitor. With respect to the calculation of the false-negative/false-positive MEP results, Blaker exact 95% confidence interval (CI) was calculated using the package “PropCIs” in the R software (R3.1.0, The R Foundation).^[13,14]^ Incidence data were compared using the chi-square test or Fisher exact test according to the expected counts. Continuous variables were compared using Student *t* test or Mann–Whitney *U* test, as appropriate. A *P* value of <0.05 was considered statistically significant.

## 3 Results

During the study period, 726 patients underwent cerebral aneurysm clipping surgery. Of the 726 patients, 41 (5.6%) were excluded from analysis because intraoperative MEP monitoring was not achieved or complete medical records were not available. Therefore, data from the remaining 685 patients (475 women and 210 men) with a mean age of 56.8 years (range, 26–82 years) were reviewed. Patients were divided into 2 groups according to the intraoperative use of NMB: no NMB group (n = 276) and partial NMB group (n = 409). Patient characteristics are shown in Table 1. The intraoperatively administered doses of propofol and remifentanil were 5.6 ± 0.9 mg/kg/h and 0.35 ± 0.08 μg/kg/min, respectively. The incidence of bradycardia requiring treatment was 28.0%, and the mean administered dose of phenylephrine was 528.5 ± 386.4 μg/h. In partial NMB group, twitch height of the first evoked response of TOF stimulation compared with the control twitch was 0.5 ± 0.1 during MEP monitoring. No incidence of bite injury to the tongue or lip was observed. Spontaneous movement or spontaneous respiration was not identified in either group. Intraoperative anesthesia-related variables are shown in Table 2. Mean MEP stimulation intensity was 265.6 ± 72.67 V. Intraoperative MEP parameters are shown in Table 3.

**Table 1:**
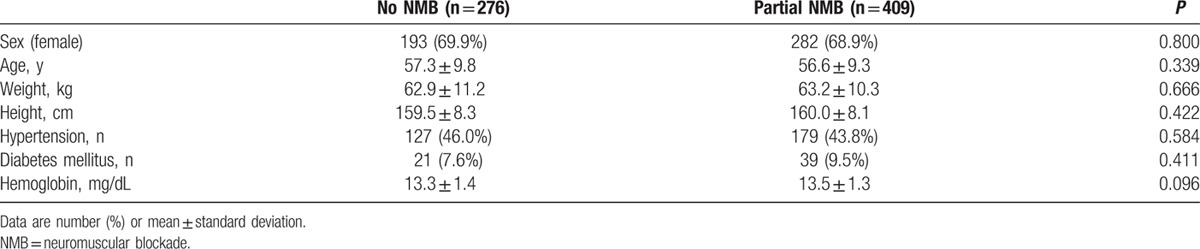
Patient demographic data.

**Table 2:**
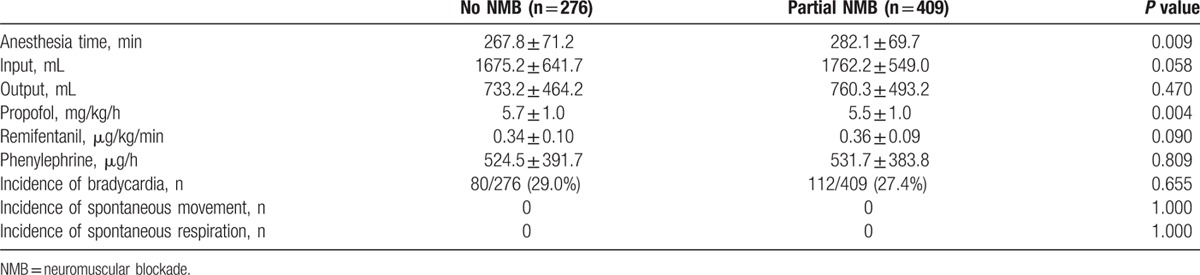
Intraoperative anesthesia related variables.

**Table 3:**
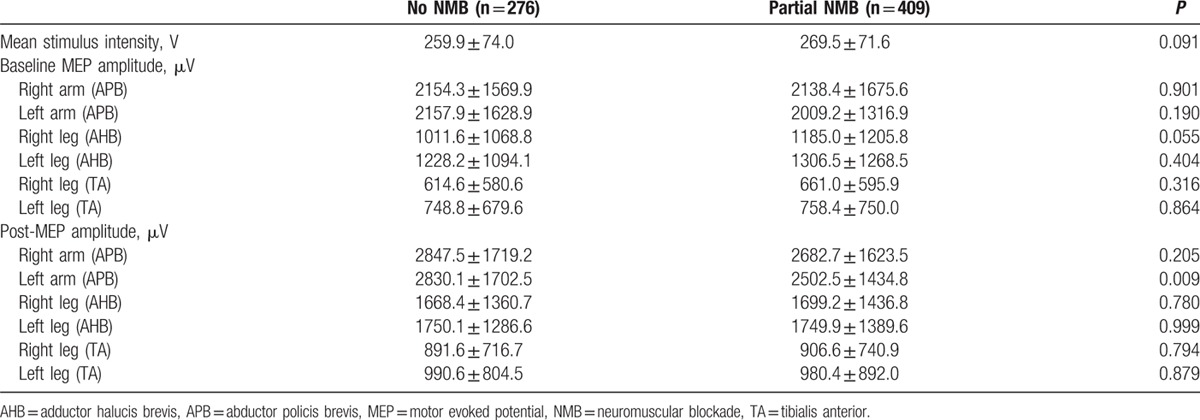
Intraoperative MEP monitoring parameters.

Table 4 describes the cases with intraoperative MEP changes and postoperative neurologic outcome. Of the 685 patients, 622 (90.8%) manifested no intraoperative changes in MEP and no postoperative motor deficits. However, 43 (6.3%) of the 685 patients showed significant intraoperative MEP changes. Of these 43 patients with significant MEP changes (irreversible or partly reversible in 13 patients and completely reversible in 30), the postoperative motor status was normal in 27 patients (62.8%). Sixteen (30%) of the 43 patients had a motor deficit, which was transient in 14 patients and permanent in 2. Twenty patients showed PNDs despite preserved intraoperative MEP, which was transient in 19 patients and permanent in 1. In the no NMB group, the sensitivity and specificity of MEP changes toward PNDs were 72.7% and 96.6%, respectively, whereas the positive predictive value (PPV) and negative predictive value (NPV) were 47.1% and 98.8%, respectively. In the partial NMB group, the sensitivity and specificity of MEP changes toward PNDs were 32.0% and 95.3%, respectively, whereas the PPV and NPV were 30.8% and 95.6%, respectively. Specifically, false-positive MEP results were found in 10 patients (3.6%; point estimate [PE], 0.0362; CI, 0.0186–0.0655) in the no NMB group and 16 (3.9%; PE, 0.0391; CI, 0.0236–0.0626) in the partial NMB group (*P* = 1.000). False-negative MEP results were found in 3 of 276 patients (1.1%; PE, 0.0109; CI, 0.0030–0.0311) in the no NMB group and 17 of 409 patients (4.2%; PE, 0.0416; CI, 0.0246–0.0651) in the partial NMB group (*P* = 0.020). False-negative rates of MEP were 27.3% (3/11) in the no NMB group and 68.0% (17/25) in the partial NMB group.

**Table 4:**
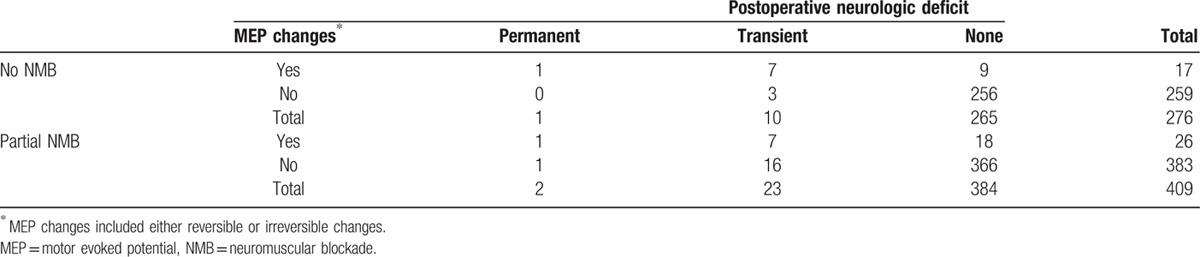
Reliability of intraoperative MEP changes for indicating the occurrence of postoperative motor deficits.

In the no NMB group, the sensitivity and specificity of irreversible MEP changes toward PNDs were 50.0% and 77.8%, respectively, whereas the PPV and NPV were 66.7% and 63.6%, respectively (Table 5). In the partial NMB group, the sensitivity and specificity of irreversible MEP changes toward PNDs were 25.0% and 72.2%, respectively, whereas the PPV and NPV were 28.6% and 68.4%, respectively. In the no NMB group, the sensitivity and specificity of reversible changes (against partly reversible and irreversible changes) in the absence of PNDs were 77.8% and 50.0%, respectively, whereas the PPV and NPV were 63.6% and 66.7%, respectively. In the partial NMB group, the sensitivity and specificity of reversible changes in the absence of PNDs were 72.2% and 25.0%, respectively, whereas the PPV and NPV were 68.4% and 28.6%, respectively.

**Table 5:**
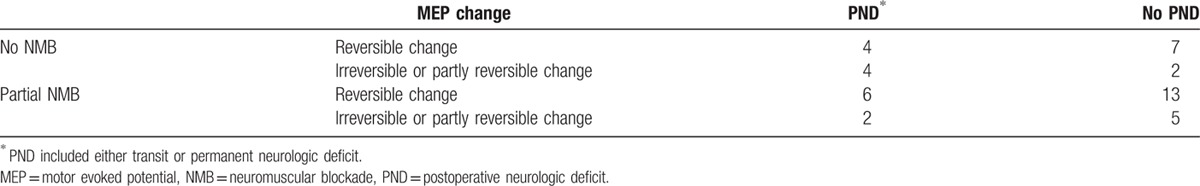
Subtype of intraoperative MEP changes and postoperative neurologic deficit.

## 4 Discussion

We compared the effect of no NMB and partial NMB on the diagnostic accuracy of MEP monitoring and unacceptable patient movement during cerebral aneurysm surgery. False-negative MEP results were significantly lesser in the no NMB group than in the partial NMB group. Furthermore, propofol/remifentanil-based anesthesia guaranteed patient immobilization not only in the partial NMB group, but also in the no NMB group. These results suggest that propofol/remifentanil-based TIVA without NMB is a safe anesthetic protocol for patients undergoing cerebral aneurysm clipping surgery, and may be helpful in enhancing the diagnostic accuracy of MEP.

We found that the sensitivity of MEP was 72.7% in the no NMB group and 32.0% in the partial NMB group. Furthermore, false-negative MEP results were significantly different between the 2 groups. Although the mechanism underlying false-negative MEP results remains to be determined, the results could likely be explained by the fact that high-intensity transcranial stimulation (up to 1500 V) can stimulate the corticospinal tract fibers even in caudal regions, such as the foramen magnum.^[15]^ Although we never used such a high-intensity stimulation during surgery, parts of other fibers might have been activated even at a stimulation intensity of 500 V.^[3]^ In our study, we reduced the stimulation intensity to around 250 V, which helped decrease the false-negative rate as compared with previous studies.^[3,6]^ The presence of NMB could necessitate a higher stimulus intensity, and partially paralyzed patients require a higher stimulation intensity.^[16]^ Very high stimulus intensity can activate the deep subcortical motor pathways and can bypass higher cortical levels, which, in turn, might lead to the generation of MEPs from the contralateral limbs despite cortical ischemia.^[2]^ The increase in MEP stimulus requirements might indicate the deepening of activation to levels below an ischemic level.^[12]^ Therefore, decreasing the stimulation intensity will enhance the diagnostic accuracy of transcranial MEP, and it is recommended that the lowest possible stimulation intensity be used.^[11,16]^ Kim et al^[6]^ reported that MEP amplitude variability significantly increased in patients with partial NMB than in those with no NMB. The increased variability and smaller amplitude may increase the incidence of false-negative MEP results, thus resulting in the relatively poor efficacy of MEP. A high-intensity stimulation may bypass a cortical lesion by stimulating the motor tract fibers distal to the lesion, thereby yielding false-negative results.^[16]^

In 27 patients, significant MEP changes were followed by an unchanged motor status. Intraoperative MEP might help detect cerebral ischemia and avoid corticospinal tract damage by enabling repositioning of the clip. MEP has been reported to reappear in 10 of 14 patients after immediate repositioning of the clip,^[5]^ and MEP recovered in 19 of 20 patients after the repositioning of the clip.^[17]^ In contrast, postoperative motor deficit occurred despite the preservation or gradual recovery of MEPs in 20 patients in our cohort. Although the underlying mechanism is still unclear, motor deficits may develop even after MEP has recovered because of the repositioning of the clip.^[3]^ In addition, the efficacy of MEP monitoring may differ according to the location of aneurysm. Studies have shown that intraoperative MEP monitoring is useful in anterior choroidal aneurysms but may yield doubtful results in middle cerebral artery (MCA) aneurysms.^[3]^ Our finding showed false-negative results for MCA and anterior choroidal aneurysms in 6 and 2 patients, respectively.

Apart from the potential influence on MEP results, the use of muscle relaxants during aneurysm clipping has several drawbacks. It increases technical complexity and introduces a potential confounding factor at critical times in the surgery.^[1]^ Furthermore, the use of NMB may not eliminate the risk of movement, and partial NMB aimed at 50% of the baseline value may elicit patient movement in response to transcranial stimulation and may interfere with surgery.^[18]^ In case of monitoring only the upper extremity, MEP signals can usually be achieved with less than 150 V of stimulation.^[16]^ However, some authors have reported difficulty in consistently obtaining MEP response in the lower extremities without using a stimulus intensity that leads to the stimulation of motor pathways below the target territories.^[11,16]^ Although increased stimulus intensity may contribute to an improved success rate in monitoring capability, it is expected to contribute to movement-related problems.^[10]^ It has been recommended that one should use the lowest stimulation intensity possible to avoid deeper activation because this reduces patient movement.^[4,11]^ In our experience, MEP monitoring of the upper and lower extremities could be achieved with approximately 250 V of stimulation. Moreover, this could be done without patient movement and, therefore, without paralyzing the patient to avoid movement.

Johnson et al^[19]^ suggested that the loss of responsiveness interaction model effectively predicted the return of responsiveness of patients during emergence from anesthesia, which was similar to patients being aware and moving while undergoing surgery. Although the target concentrations of propofol and remifentanil that can produce 100% unresponsiveness during laryngoscopy were not presented, there may be a more than 95% probability of no response at an approximate propofol concentration of 3.0 μg/mL and a remifentanil concentration of 12 ng/mL.^[19]^ In addition, a higher effect-site concentration of remifentanil lessens the risk of movement in the absence of muscle relaxants with surgical stimulation for craniotomy.^[20]^ The TIVA technique was significantly more effective at preventing movement intraoperatively.^[21]^ Only 3% of patients in the TIVA group moved, whereas more than 28% in the desflurane group experienced movement.^[21]^ Bispectral index and electroencephalogram entropy are more effective at predicting unconsciousness than movement intraoperatively.^[22]^ Movement may be related to subcortical function rather than to cortical activity. Rampil et al^[23]^ showed that the minimum alveolar anesthetic concentration was similar in decerebrated animals and controls. As a higher dose of remifentanil lessens the risk of movement during craniotomy in the absence of NMB,^[20]^ TIVA may be suitable for patients undergoing cerebral aneurysm clipping surgery with MEP monitoring. Based on the response surface model, 0.5 minimum alveolar concentration of a volatile anesthetic combined with 0.19 μg/kg/min of remifentanil infusion is required to obtain a more than 95% probability of immobilization.^[24]^ However, increasing the dose of propofol is not appropriate, because the MEP amplitude can be suppressed by propofol in a dose-dependent manner.^[25,26]^

Proponents of partial NMB insist that the increasing depth of anesthesia required to avoid movement in nonparalyzed patients may cause bradycardia and hypotension.^[27]^ In our study, the overall incidence of bradycardia (28%) was relatively higher than that in a previous study (9.1%).^[6]^ However, there was no difference in the incidence of bradycardia between the partial NMB group and the no NMB group. Moreover, most cases of bradycardia were successfully treated by bolus administration of vagolytic agents. Intraoperative hypotension can be managed with continuous infusion of approximately 500 μg/h of phenylephrine. Studies have shown that phenylephrine increases cerebral blood flow by increasing the cerebral perfusion pressure.^[28]^ Phenylephrine is the most commonly used vasopressor in neuroanesthesia and neurocritical care units.^[29]^

There are several limitations to this study. First, the retrospective nature of the study entails a set of limitations. Rates of motor deficits obtained from the review of medical records may be underestimated because of the varying quality of information in the recorded entries. However, we believe our current analysis offers valuable clinical information for readers who are involved in neurocritical care. Second, hyperalgesia or acute tolerance, which is one of the serious concerns after high-dose remifentanil infusion, was not evaluated. However, the mean intraoperative infusion dose in our study cohort (0.35 μg/kg/min) was relatively lower than the dose associated with acute tolerance (0.6–0.9 μg·kg/min).^[30]^ Third, we evaluated the diagnostic efficacy of MEP monitoring by using arbitrary criteria, even though there are various criteria to enumerate PNDs and to determine abnormal MEP results. Irie et al^[3]^ reported that the false-negative results in MEP monitoring may include new-onset hemiparesis, but we did not consider delayed PNDs. Nevertheless, other criteria regarding MEP changes can be implemented, including alteration in MEP morphology, changes in MEP amplitude, and changes in threshold-level variables. Further studies are needed to evaluate the diagnostic efficacy of multimodal electrophysiologic monitoring including somatosensory EP under various anesthetic regimens.

In conclusion, propofol/remifentanil-based TIVA without NMB facilitates a decrease in the stimulation intensity of MEP, which may decrease the false-negative ratio of MEP monitoring during cerebral aneurysm surgery. Our anesthetic protocol provided not only reliable intraoperative MEP recording, but also patient immobilization during cerebral aneurysm clipping surgery.
